# Feeding Rhythm-Induced Hypothalamic Agouti-Related Protein Elevation via Glucocorticoids Leads to Insulin Resistance in Skeletal Muscle

**DOI:** 10.3390/ijms221910831

**Published:** 2021-10-07

**Authors:** Tetsuya Shiuchi, Airi Otsuka, Noriyuki Shimizu, Sachiko Chikahisa, Hiroyoshi Séi

**Affiliations:** 1 Department of Integrative Physiology, Institute of Biomedical Sciences, Tokushima, University Graduate School, Tokushima 770-8503, Japan; c201556020@tokushima-u.ac.jp (A.O.); shimizun@tokushima-u.ac.jp (N.S.); chika@tokushima-u.ac.jp (S.C.); sei@tokushima-u.ac.jp (H.S.); 2 Precursory Research for Embryonic Science and Technology, Japan Science and Technology Agency, Kawaguchi 332-0012, Japan

**Keywords:** AgRP, glucocorticoid, feeding rhythm, insulin sensitivity, skeletal muscle

## Abstract

Circadian phase shifts in peripheral clocks induced by changes in feeding rhythm often result in insulin resistance. However, whether the hypothalamic control system for energy metabolism is involved in the feeding rhythm-related development of insulin resistance is unknown. Here, we show the physiological significance and mechanism of the involvement of the agouti-related protein (AgRP) in evening feeding-associated alterations in insulin sensitivity. Evening feeding during the active dark period increased hypothalamic AgRP expression and skeletal muscle insulin resistance in mice. Inhibiting AgRP expression by administering an antisense oligo or a glucocorticoid receptor antagonist mitigated these effects. AgRP-producing neuron-specific glucocorticoid receptor-knockout (AgRP-GR-KO) mice had normal skeletal muscle insulin sensitivity even under evening feeding schedules. Hepatic vagotomy enhanced AgRP expression in the hypothalamus even during ad-lib feeding in wild-type mice but not in AgRP-GR-KO mice. The findings of this study indicate that feeding in the late active period may affect hypothalamic AgRP expression via glucocorticoids and induce skeletal muscle insulin resistance.

## 1. Introduction

Lifestyle-related diseases have been linked to obesity, type 2 diabetes, lipodystrophy, heart disease, and cancer, and have become one of the most serious health problems of our time. In particular, dietary habits are a major cause of lifestyle-related diseases, such as peripheral artery disease and obesity [1,2], and need to be reviewed to extend our healthy life expectancy. Obesity mainly occurs as a result of energy intake constantly exceeding energy consumption. 

Whole-body energy metabolism is reportedly influenced by circadian rhythms, which comprise light-dark and feeding rhythms [3,4]. The circadian clock system plays an important role in maintaining physiological functions [5]. The molecular mechanisms of this system are well studied in rodents [6]. The disruption of circadian oscillator components in the suprachiasmatic nucleus of the hypothalamus, which is master regulator of circadian rhythm in mammal, causes internal desynchronization in rodents and is considered a risk factor for metabolic disorders including obesity and diabetes. Several studies have reported the synchronization of peripheral clocks to different feeding patterns [7,8,9,10,11]. Shifts in hepatic clock gene expression patterns disturb feeding rhythms, contributing to changes in hepatic lipid metabolism and consequent whole-body insulin resistance. The feeding rhythm is stronger than habitual exercise for the prevention of diet-induced obesity [12]. These observations indicate that feeding schedules affect both metabolic states and circadian rhythms in rodents, but it remains unknown whether feeding rhythms influence the hypothalamic control system for energy metabolism.

The hypothalamus is the control tower for maintaining energy balance in an individual, controlling feeding behavior, and regulating insulin sensitivity in peripheral tissues, such as skeletal muscle and liver [13,14,15,16,17,18,19,20,21,22]. Various neuropeptides are abundantly expressed in the hypothalamus, and their actions cause elaborate energy metabolism regulation [23,24]. Humoral signals regulate hypothalamic neuropeptides from peripheral tissues via neural or bloodstream [23]. Inter-tissue communication about energy metabolism is required to balance the energy demands of the body with supply [25]. However, the effects of feeding rhythm on hypothalamic neuropeptide expression and action and its mechanism have not been clarified. It is well known that the clock genes in the liver shift phases due to feeding rhythm [9,10,11]. It has also been reported that there is a neurological connection between the liver and the brain [25,26] and that it acts as a pathway that regulates peripheral energy metabolism. These previous studies suggest that feeding rhythm may indirectly change the metabolic regulation mechanism of the hypothalamus.

Thus, we hypothesized that feeding rhythm alters gene expression in hypothalamus, which regulates energy homeostasis via peripheral-central communication. To test this hypothesis, we aimed to identify the gene in hypothalamus responsible for fluctuations in energy metabolism due to changes in feeding rhythm and to find a peripheral pathway that regulates the hypothalamic gene.

## 2. Results

### 2.1. Evening-Type Feeding Rhythm Leads to Skeletal Muscle Insulin Resistance

**Figure 1:**
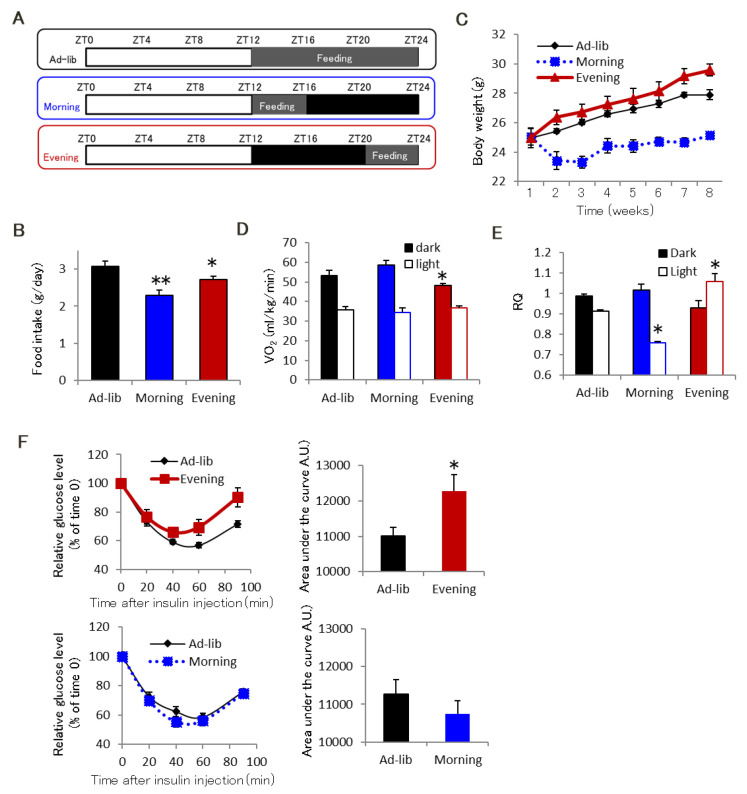
Evening feeding leads to whole body insulin resistance. (**A**) Feeding schedule for this study. (**B**) Food intake in the Morning and Evening groups was lower than in the Ad-lib group (*n* = 8–11). (**C**) Body weight in the Ad-lib and Evening groups, but not the Morning group, gradually increased (*n* = 8–11). (**D**,**E**) Respiratory analysis indicated that the Evening group (*n* = 6) had decreased VO_2_ (**D**) and increased RQ (**E**). (**F**) Relative glucose levels (left) after injection of insulin (1 U/kg) in the Evening group (ZT4, upper), in the Morning group (ZT20, bottom) with the respective Ad-lib group. The Evening group (*n* = 6) had a significantly greater area under the curve (right) than the Ad-lib group. Right panels are the area under the curve of respective ITT. Data are presented as mean ± standard error. * *p* < 0.05, ** *p* < 0.01 vs. Ad-lib group.

We used the three feeding schedules shown in Figure 1A. Mice were fed ad libitum during either the dark active period (ZT12-24, Ad-lib group), the first 4 h of the dark phase (ZT12-16, Morning group), or the last 4 h of the dark phase (ZT20-24, Evening group). Food intake in the Morning and Evening groups was significantly lower than that in the Ad-lib group (Figure 1B). Body weight in the Morning group did not increase for 8 weeks and was lower than that in other groups (Figure 1C). Despite lower food intake, the Evening group had body weight gains similar to those observed in the Ad-lib group (Figure 1C). The Evening group had lower oxygen consumption (VO_2_) and a higher respiratory quotient (RQ) than the Ad-lib group (Figure 1D,E). On the 8th week, we performed an insulin tolerance test (ITT) 4 h after fasting in the Morning (ZT20) and Evening (ZT4) groups. We performed the ITT at a comparable time point in the Ad-lib group. Insulin tolerance was similar in the Morning and Ad-lib groups but lower in the Evening group (Figure 1F). The Evening group had significantly higher fasting plasma glucose (101.11 ± 5.99 mg/dL in Ad-lib, 115.08 ± 4.06 mg/dL in Evening, *p* < 0.05) and insulin (0.31 ± 0.03 ng/mL in Ad-lib, 0.89 ± 0.13 ng/mL in Evening, *p* < 0.01) levels than the Ad-lib group. In addition, we performed glucose tolerance test (GTT) and observed that glucose tolerance remains unaltered in the Ad-lib and Evening groups. However, plasma insulin level was significantly higher in the Evening group than in the Ad-lib group (Figure S1), indicating that glucose tolerance in the Evening group was improved by enhanced insulin secretion. These results suggest that an evening feeding schedule for 8 weeks leads to whole-body insulin resistance. 

**Figure 2:**
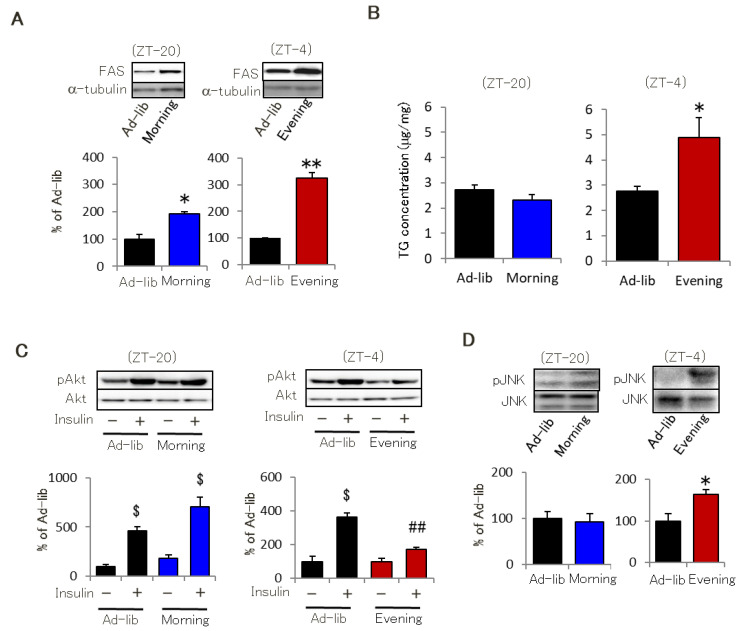
Evening feeding leads to skeletal muscle insulin resistance. (**A**) FAS expression in soleus muscle (*n* = 4). (**B**) TG content in gastrocnemius muscle (*n* = 6–8). (**C**) Akt phosphorylation in soleus muscle with or without insulin injection (*n* = 3–4). Insulin was injected at same time point of ITT. (**D**) JNK phosphorylation in the soleus (*n* = 3–4). Data are presented as mean ± standard error. * *p* < 0.05, ** *p* < 0.01 vs. Ad-lib group. ^$^ *p* < 0.05 vs. Akt phosphorylation of each group without insulin. ^##^ *p* < 0.01 vs. Ad-lib group (insulin +).

Previous research indicates that disturbed feeding rhythms influence the circadian expression of hepatic clock genes that then impair energy metabolism associated with hepatic lipid accumulation. Therefore, we investigated the circadian expression of clock genes and triglyceride (TG) content in the liver. Consistent with previous reports, the Evening group exhibited different circadian expression patterns for hepatic clock genes than the other two groups (Figure S2A–D), but the groups exhibited similar hepatic fatty acid synthase (FAS) expression levels and TG content (Figure S2E,F). In contrast, the Evening group showed significantly higher FAS expression levels and TG content in skeletal muscle than the other groups. However, the Morning group also exhibited higher FAS expression than the Ad-lib group (Figure 2A,B). Intraperitoneal (i.p.) insulin injections increased skeletal muscle Akt phosphorylation in the Ad-lib group (Figure 2C). The Morning group showed a similar increase in phosphorylation, while the Evening group exhibited lower Akt phosphorylation levels (Figure 2C). IP injection for insulin-induced Akt phosphorylation in the liver was similar in the three groups (Figure S2G). The skeletal muscles of the Evening group increased phosphorylation of Janus kinase (JNK), which is enhanced by lipid accumulation and inhibits insulin signaling, and exhibited reduced phosphorylation of adenosine monophosphate kinase (AMPK), which is involved in fatty acid oxidation (Figure 2D and data not shown). The three groups did not significantly differ in muscle mass (data not shown). These results show that evening feeding leads to insulin resistance due to impaired skeletal muscle insulin sensitivity and lipid accumulation.

### 2.2. AgRP Is Responsible for Evening-Type Feeding Rhythm-Induced Insulin Resistance

**Figure 3:**
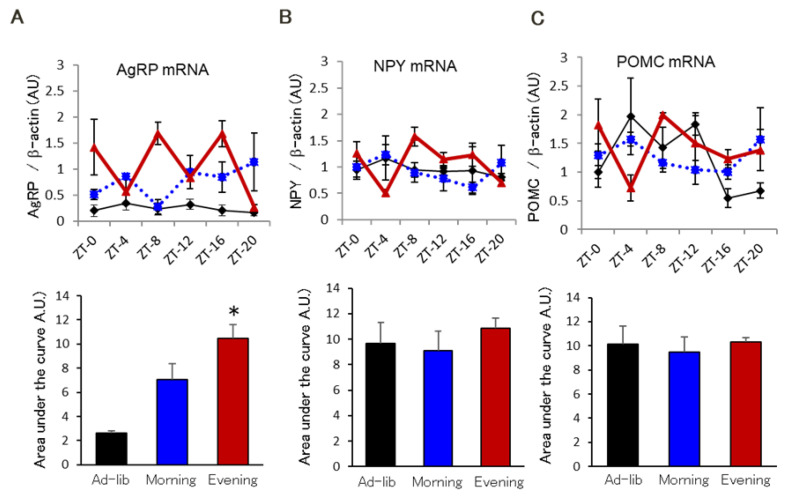
AgRP is a candidate for the evening feeding rhythm-induced insulin resistance. Real time quantitative PCR in 24-h (*n* = 3–4). (**A**) AgRP (**B**) NPY (**C**) POMC. Red, blue, and black lines represent the Evening, Morning, and Ad-lib groups, respectively. Bottom panel shows the area under the curve of each gene mRNA circadian expression. * *p* < 0.05 vs. Ad-lib group.

To investigate the effects of the feeding schedule on the expression of hypothalamic molecules, we performed microarray analysis. We found that the Evening group had a more than 2-fold increase in hypothalamic gene expression relative to the Morning and Ad-lib groups. Fifteen genes were upregulated in the hypothalamus in Evening group (Table S1). The most upregulated gene encodes for Agouti-related protein (AgRP). Quantitative real-time polymerase chain reaction (qPCR) indicated that the Evening group showed continuously higher circadian mRNA expression of AgRP than the other groups (Figure 3A). In contrast, the groups exhibited similar expression levels of NPY (Figure 3B) and pro-opiomelanocortin (POMC) (Figure 3C), which, like AgRP, are expressed in the arcuate nucleus of the hypothalamus. However, these molecules were unbound during the circadian expression phase (Figure 3A–C). Interestingly, glucocorticoid regulated kinase 1 (SGK1), which is tightly regulated by glucocorticoid and serum was upregulated in the hypothalamus of the Evening group (Table S1). 

To check whether AgRP is responsible for the feeding rhythm-induced insulin resistance of the Evening group, we injected AgRP into the lateral ventricles for 1 week. AgRP-injected mice pair-fed with saline-injected mice had increased RQ and decreased whole-body VO_2_ and insulin-induced skeletal muscle Akt phosphorylation (Figure S3A–C). Additionally, AgRP-injected mice tended to have an increased TG content and skeletal muscle JNK phosphorylation (Figure S3D,E). Furthermore, single AgRP injections decreased norepinephrine (NE) turnover, indicating sympathetic nerve activity in skeletal muscle (Figure S3F). Sciatic denervation, which disrupts sympathetic nerve projections to skeletal muscle, increased FAS expression (Figure S3G). Injecting AgRP into denervated mice did not further enhance FAS expression (Figure S3G). These results indicate that the cerebral action of AgRP leads to similar metabolic phenomena with the Evening group partly mediated by sympathetic nerve activity reduction.

**Figure 4:**
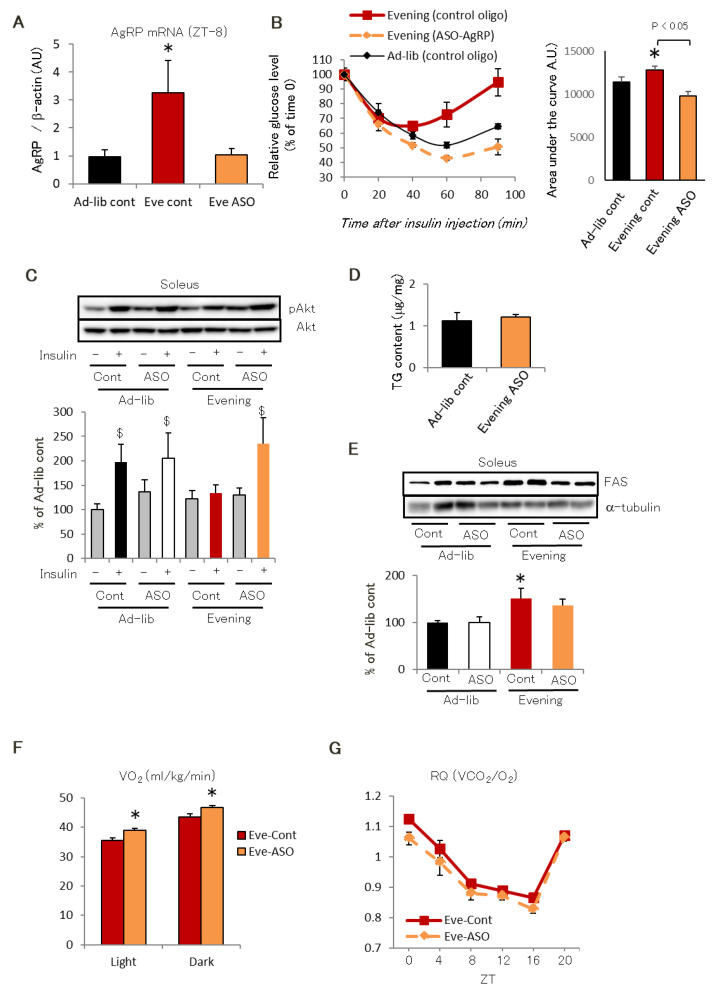
AgRP is responsible for the evening feeding rhythm-induced insulin resistance. (**A**) AgRP mRNA expression in the hypothalamus after intracerebroventricular antisense oligonucleotide (ASO) administration (twice a week) in the Evening group. (**B**) insulin tolerance test (ITT). (**C**–**E**) Akt phosphorylation in soleus (*n* = 3) (**C**), TG content in gastrocnemius (*n* = 6) (**D**), FAS expression in soleus (**E**) following ASO treatment. (**F**,**G**) VO_2_ in the dark and light period (**F**) and circadian RQ over 24 h (*n* = 6) following ASO treatment in the each Evening group. Data are presented as mean ± standard error. * *p* < 0.05 vs. Ad-lib group. ^$^ *p* < 0.05 vs. Akt phosphorylation of each group without insulin.

Thus, we gave the Evening group injections of an antisense oligonucleotide against AgRP twice-weekly to reduce AgRP expression [27]. Eight weeks later, the Evening group displayed similar effects to those in the Ad-lib group following reduced AgRP expression, including decreased whole-body insulin tolerance, TG content, and FAS expression and increased insulin-induced skeletal muscle Akt phosphorylation (Figure 4A–E). Moreover, in the Evening group, the RQ was decreased within a day, while VO_2_ reductions were reversed (Figure 4F,G). These findings suggest that hypothalamic AgRP was responsible for the evening feeding schedule-induced insulin resistance in skeletal muscle.

### 2.3. Glucocorticoid Sensitivity in the Hypothalamus Is Critical for AgRP Expression Due to the Evening-Type Feeding Rhythm

**Figure 5:**
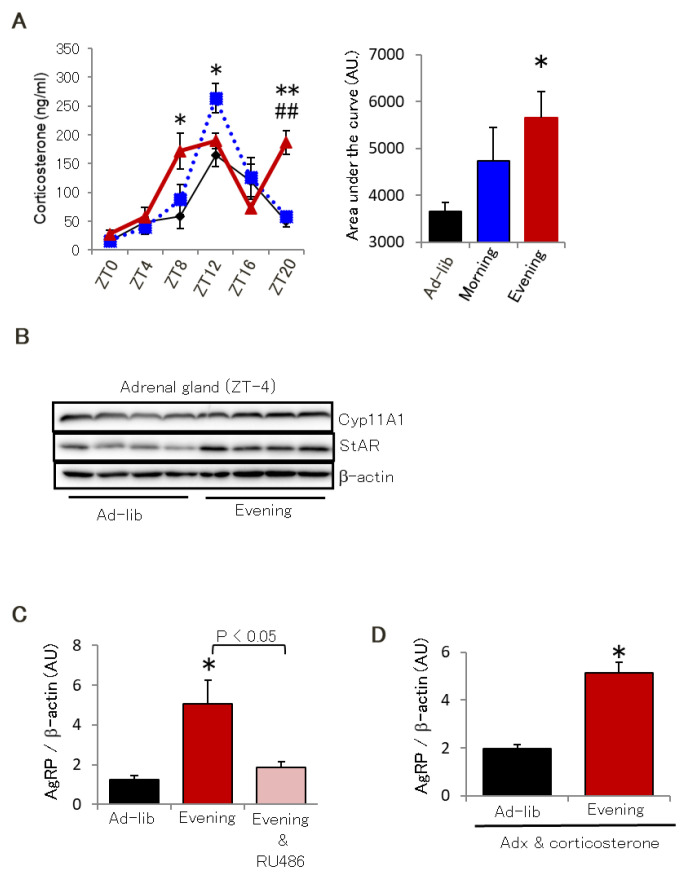
Hypo thalamic glucocorticoid sensitivity is critical for evening feeding-induced AgRP expression. (**A**) Circadian rhythm of plasma corticosterone levels (left) and areas under the curve (right) (*n* = 4). (**B**) Two enzymes expression required for glucocorticoid synthesis, Cyp11a1 and StAR, in the adrenal glands (*n* = 4). (**C**) Effect of oral administration of the glucocorticoid receptor antagonist RU-486 (20 mg/kg/day) for 2 weeks on AgRP expression of the hypothalamus (*n* = 5). (**D**) Effect of adrenalectomy and 2 weeks of corticosterone supplementation (Adx) on AgRP expression of the hypothalamus (*n* = 5). Data are presented as mean ± standard error. * *p* < 0.05 vs. Ad-lib group. ** *p* < 0.01 vs. Ad-lib group. ## *p* < 0.01 vs. Morning group.

To clarify the mechanism of elevated AgRP expression following evening feeding, we tried to identify critical nutrient factors in plasma, as AgRP is regionally expressed in the hypothalamic arcuate nucleus, where the blood-brain barrier is leaky. We measured plasma glucose, TG, non-esterified fatty acids, cholesterol, total bile acid, insulin, individual fatty acids, and amino acids. However, we did not identify any nutrient responsible for our observations (Figure S4 and data not shown). We also focused on the hormone levels previously reported to be involved in AgRP expression. Corticosterone secretion has a circadian rhythm and stimulates AgRP expression [28,29]. As shown in Figure 5A, the Evening group had disturbed circadian rhythms of plasma corticosterone and larger areas under the curve. Moreover, the Evening group showed increased Cyp11a1 and StAR expression, two enzymes necessary for glucocorticoid synthesis in the adrenal gland (Figure 5B). This suggests that the Evening group had a disturbed circadian rhythm and a higher daily corticosterone exposure time than the other two groups. Oral administration of the glucocorticoid receptor (GR) antagonist RU-486 for 2 weeks significantly reversed the elevated AgRP expression of the Evening group (Figure 5C). We thus performed adrenalectomy followed by continuous corticosterone infusion with an osmotic mini-pump for 2 weeks to compensate for the corticosterone exposures of the groups, but we could not inhibit the increased AgRP expression in the Evening group (Figure 5D). These results suggest that evening feeding might enhance glucocorticoid sensitivity, especially in AgRP-producing neurons.

### 2.4. AgRP Neuron-Specific GR Knockout Mice Have Normal Insulin Sensitivity Even in the Evening-Type Feeding Rhythm

**Figure 6:**
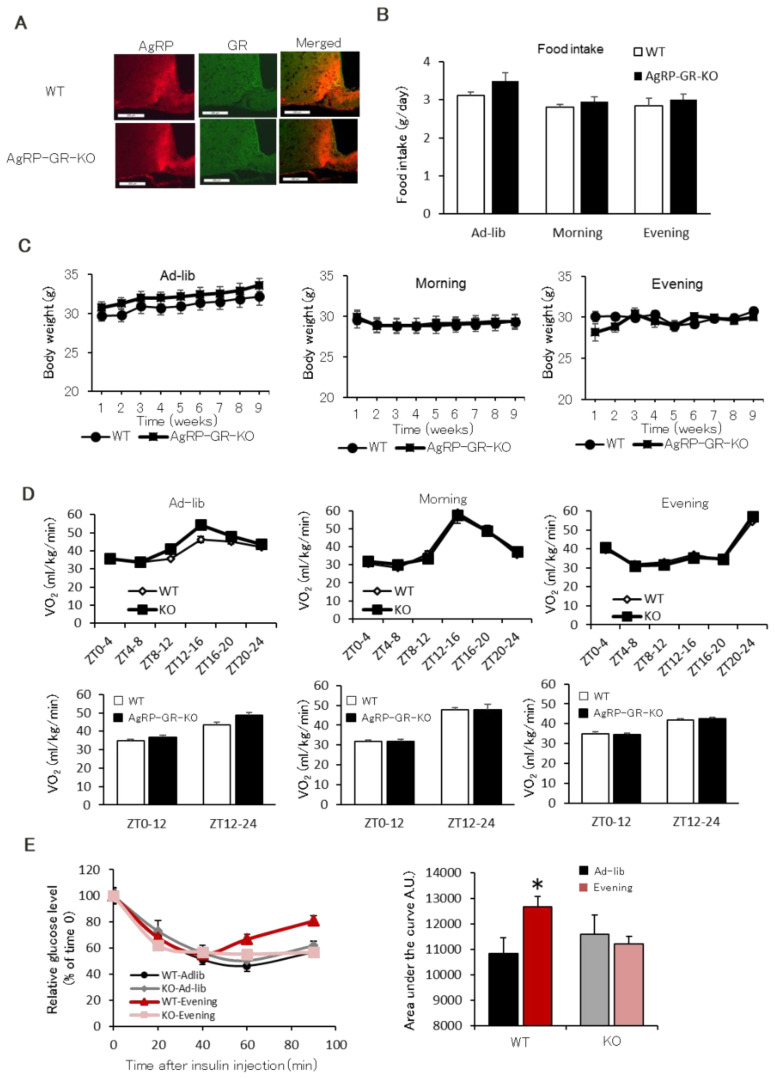
AgRP neuron-specific GR deficient mice has no insulin tolerance even in the Evening feeding schedule. (**A**) Immunohistochemistry of the hypothalamic arcuate nucleus in WT and AgRP-GR-KO mice. The scale bars indicate 100 μm. (**B**) Food intake in WT and AgRP-GR-KO mice. (**C**) Alteration of body weight in WT and AgRP-GR-KO mice. (**D**) Circadian change of oxygen consumption in WT and AgRP-GR-KO mice. White column shows light period, black column shows dark period of VO_2_ in bottom panels. (**E**) Insulin tolerance in Evening group WT and AgRP-GR-KO mice (*n* = 6). * *p* < 0.05 vs. Ad-lib group.

As shown in Figure 5D, we established that AgRP expression could be suppressed by administering an inhibitor of the glucocorticoid receptor. However, this drug acts on the whole body and directly on skeletal muscle. Therefore, even if we examine the insulin sensitivity, we cannot precisely affirm whether this effect is due to the drug or AgRP suppression. Since it was known that AgRP is upregulated by glucocorticoids, we assumed that the above question could be addressed if its receptor GR, could be specifically deleted in AGRP neurons. Therefore, to confirm the role of the glucocorticoid-AgRP pathway in insulin resistance observed in the evening feeding schedule group, we established AgRP neuron-specific GR knockout (AgRP-GR-KO) mice using a Cre-loxP system (Figure 6A). We placed wild-type (WT) and AgRP-GR-KO mice on the three feeding schedules as described in Figure 1A and reared them for 8 weeks. Like WT mice, AgRP-GR-KO mice showed lower food intake in the Morning and Evening group than in the Ad-lib group (Figure 6B). Change in body weight for 8 weeks was similar between WT and AgRP-GR-KO mice (Figure 6C). The circadian rhythm of VO_2_ differed among three groups, while average and circadian rhythm of VO_2_ in each group showed no difference between WT and AgRP-GR-KO mice (Figure 6D). On the contrary, AgRP-GR-KO mice had high RQ especially during the light period of the evening feeding schedule (Figure S5). ITT showed that whole-body insulin sensitivity was reduced in the Evening group of WT mice. In contrast, AgRP-GR-KO mice did not reduce whole-body insulin sensitivity even in the Evening group (Figure 6E).

**Figure 7:**
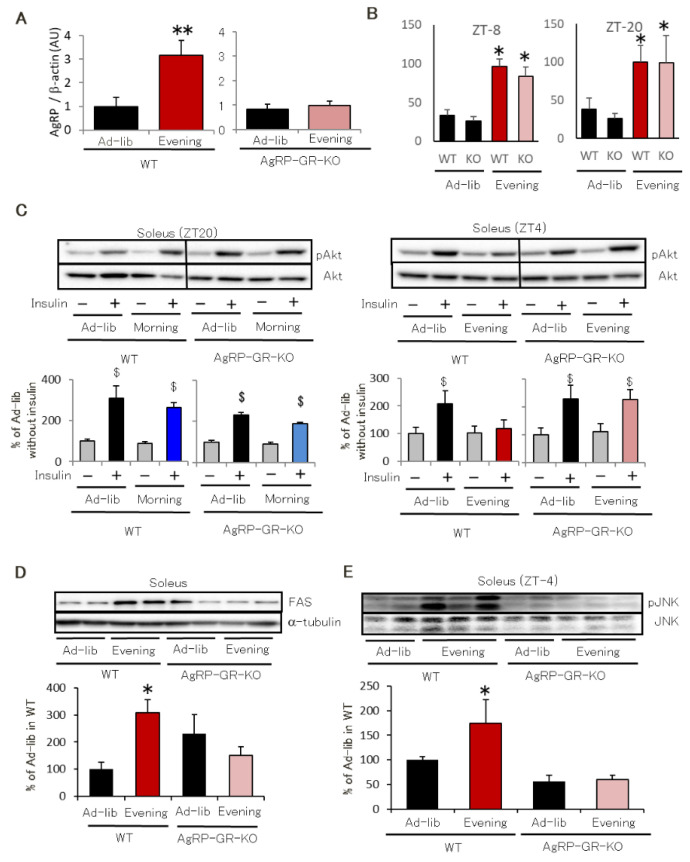
AgRP neuron-specific GR deficient mice does not show insulin resistance in skeletal muscle even in the Evening feeding schedule with decrease in AgRP expression. (**A**) Hypothalamic AgRP expression in WT and AgRP-GR-KO mice (*n* = 6). (**B**) Plasma glucocorticoid level in WT and AgRP-GR-KO mice (*n* = 6). (**C**) Insulin-induced Akt phosphorylation in the soleus in WT and AgRP-GR-KO mice (*n* = 3–5). (**D**) FAS expression in the soleus in WT and AgRP-GR-KO mice in the Ad-lib and Evening groups (*n* = 3–5). (**E**) JNK phosphorylation in soleus of WT or AgRP-GR-KO mice (*n* = 3–4). Data are presented as mean ± standard error. * *p* < 0.05, ** *p* < 0.01 vs. Ad-lib group. ^$^ *p* < 0.05 vs. Akt phosphorylation of each group without insulin.

Evening feeding for 2 weeks increased hypothalamic AgRP expression in WT mice but not in AgRP-GR-KO mice (Figure 7A). Both WT and AgRP-GR-KO mice showed higher plasma corticosterone levels in the Evening group (Figure 7B). Eight weeks into the feeding schedule, the Evening group WT mice showed lower insulin-induced Akt phosphorylation. In contrast, the AgRP-GR-KO mice did not exhibit reduced skeletal muscle Akt phosphorylation even under evening feeding (Figure 7C). The Ad-lib and Morning groups exhibited similar insulin-induced Akt phosphorylation in the soleus (Figure 7C). The AgRP-GR-KO mice did not exhibit enhanced FAS expression or JNK phosphorylation in the soleus even under evening feeding (Figure 7D,E). These results indicate that glucocorticoids directly stimulate AgRP expression in evening-fed mice, leading to insulin resistance. 

### 2.5. Hypothalamic Glucocorticoid Sensitivity Is Enhanced by Hepatic Afferent Nerve in the Evening-Type Feeding Rhythm

**Figure 8:**
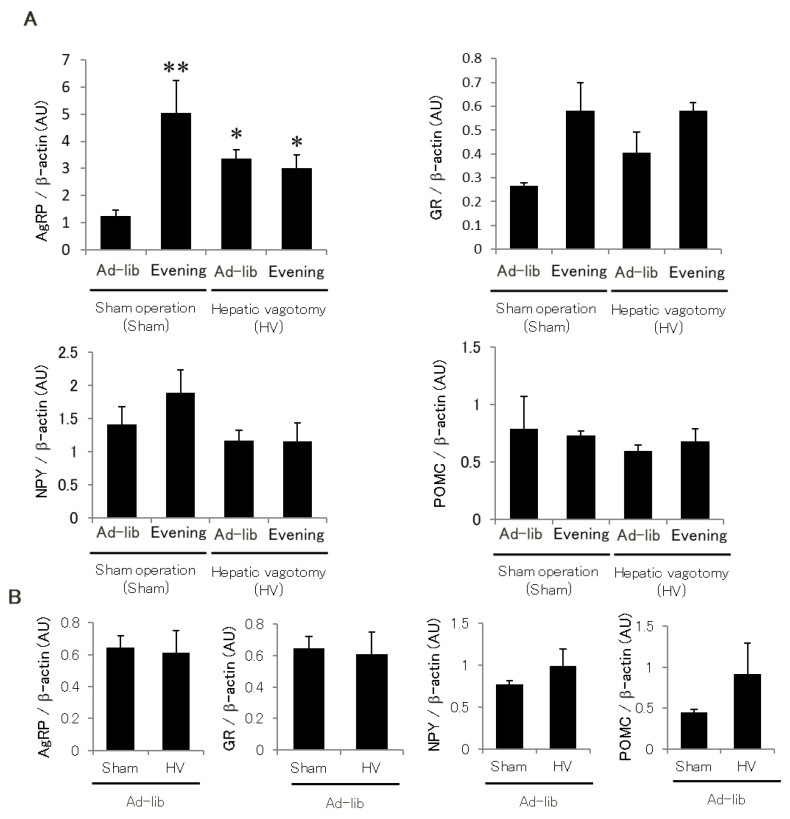
Effect of hepatic vagotomy on AgRP expression in (**A**) WT and (**B**) AgRP-GR-KO mice. (**A**) Hypothalamic AgRP, NPY, POMC and GR expression in WT mice with or without hepatic vagotomy (*n* = 5). (**B**) Hypothalamic AgRP, NPY, POMC, and GR expression in AgRP-GR-KO mice in the Ad-lib group with or without hepatic vagotomy (*n* = 5). Data are presented as mean ± SE, *n* = 4. * *p* < 0.05, ** *p* < 0.01 vs. Ad-lib (Sham).

To further investigate the mechanisms underlying hypothalamic glucocorticoid sensitivity, we focused on the hepatic afferent nerve, as the circadian phases of the clock gene shift drastically in the liver (Figure S1A–D). The Ad-lib group exhibited increased AgRP expression after hepatic vagal afferent denervation. However, hepatic vagal deafferentation did not reduce elevated AgRP and GR expression of the Evening group but not other neuropeptides in the arcuate hypothalamus such as NPY and POMC (Figure 8A). This phenomenon was not observed in AgRP-GR-KO mice with similar corticosterone levels (Figure 8B), suggesting that glucocorticoid sensitivity in AgRP neurons may mediate the decrease in hepatic vagal afferent nerve activity.

## 3. Discussion

Feeding rhythm disturbances are recognized risk factors for insulin resistance. However, many studies have focused on feeding effects in the non-active phase rather than on the central regulatory system of energy metabolism. Here, we show that feeding in the late active phase (the Evening group) might induce insulin resistance in the whole body and skeletal muscles without hyperphagia and obesity. These phenomena are mediated via the glucocorticoid-hypothalamic AgRP pathway. Eating predominantly in the evening and night is known as the night-eating syndrome and leads to insulin resistance, often without hyperphagia or obesity [30,31]. Our data in the Evening group might therefore mimic the symptoms of night eating syndrome. The feeding rhythm directly affects hepatic clock gene expression and, thus, influences hepatic and systemic lipid metabolism [7,32,33], but we found no significant differences in hepatic TG content among the three groups. One explanation for this is that the 4 h feeding restriction led to insufficient daily food intake to increase hepatic triglyceride levels. This kind of restricted feeding is known to generally induce changes similar to those found in the Morning group [7,32,33], but the Evening group developed insulin resistance without body weight reduction despite an identical feeding duration. 

Although there was no difference in TG content of liver among the three groups, TG content in skeletal muscle was higher in the Evening group than that in other groups. TG accumulation in skeletal muscle induces insulin resistance, especially in type 1 muscle [34]. Moreover, TG accumulation in skeletal muscle stimulates JNK activation, impeding insulin signaling [35,36]. In this study, Evening feeding showed decreased insulin-induced Akt phosphorylation in skeletal muscle with increased JNK phosphorylation, FAS expression, and TG accumulation. These results indicated that whole-body insulin resistance of the Evening group was mediated by decreased muscle insulin signaling via TG accumulation. 

AgRP is an important neuropeptide for feeding behavior because it antagonizes melanocortin receptors. However, it is unclear whether hypothalamic AgRP mediates insulin sensitivity in skeletal muscle. We previously reported that injecting melanocortin receptor agonists into the lateral ventricle or the ventromedial hypothalamus stimulates AMPK phosphorylation and glucose uptake in skeletal muscle [17,19]. These changes were associated with sympathetic nerve activation (data not shown). Injecting AgRP into the lateral ventricle for 1 week caused a range of sympathetic nerve-related changes in skeletal muscles, including reduced oxygen consumption, fatty acid oxidation, and insulin sensitivity, and increased FAS expression and triglyceride content. Similar changes were observed in the Evening group mice. In addition, previous reports indicated that although AgRP-KO and WT mice do not differ in terms of energy expenditure [37], that old AgRP-KO mice have increased energy metabolism [38], and that late-onset deletion of AgRP-producing neurons leads to increased sympathetic nerve activity and lipid utilization in the soleus [39]. Indeed, our study showed that antisense oligonucleotides against AgRP reduced insulin resistance in the Evening group, suggesting that hypothalamic AgRP mediates evening feeding-induced impairment of muscular insulin sensitivity. 

Chronic exposure to glucocorticoids induces several pathogenic changes, including neural damage, insulin resistance, and diabetes. GRs reportedly bind to the AgRP promoter and stimulate AgRP expression during fasting [27,40,41]. This suggests that glucocorticoids directly dysregulate AgRP. A recent report showed that female mice lacking GRs specific for AgRP-producing neurons gained less weight on a high-fat diet [42]. We also established that deleting these same GRs in male mice using a Cre-loxP system, which was different from the previous report [42], inhibits evening feeding-induced AgRP expression. Although the hepatic afferent-derived enhancement of GR sensitivity in AgRP-producing neurons contributes to AgRP expression, high glucocorticoid exposure may also contribute to the above phenomena. Moreover, exposure to high glucocorticoid levels might not directly affect muscle insulin sensitivity, as AgRP-GR-KO mice in the Evening group had normal insulin sensitivity and plasma glucocorticoid levels similar to those of WT mice in the Evening group. On the contrary, it was found that AgRP-GR-KO mice had high RQ especially during the light period of the evening feeding schedule (Figure S5), demonstrating higher carbohydrate metabolism due to improved insulin sensitivity. Since the rise in the latter half of ITT is suppressed, the GR-AgRP system may have a role in triggering sugar release. In that case, although this system did not affect hepatic insulin sensitivity, it may be involved in glucose release. We wish to pursue this research concept in future.

We observed shifts in hepatic clock gene expression and plasma glucocorticoid levels in the Evening group compared to the other groups. The hepatic afferent vagal nerve is an important information pathway for the peripheral-central metabolic relay [25,43]. Both Ad-lib and Evening groups of WT mice with vagotomized hepatic branches had increased AgRP expression, but this was not observed in AgRP-GR-KO mice. Moreover, higher AgRP mRNA expression was maintained in the Evening group by equalizing glucocorticoid levels and circadian phases using adrenalectomy and corticosterone supplementation with an osmotic mini pump. These observations suggest that information may be transferred from the liver to the hypothalamus via the afferent nerve. This information may be used to modulate GR sensitivity indirectly in AgRP-producing neurons. We further speculated that AgRP-producing neurons receive continuous afferent inhibitory input from the liver. This information may be partly regulated by the circadian hepatic expression of clock genes, which is shifted following feeding rhythm disturbances. Moreover, corticosterone regulates the synaptic input of AgRP-producing neurons [44], implying that enhanced GR sensitivity in AgRP neurons in the evening feeding schedule increases AgRP neuronal activity with an increase in synaptic input. Additionally, even if the insulin sensitivity of the liver remains unchanged, metabolic fluctuations due to phase changes in clock genes may occur. It is speculated that part of metabolites acts on the hepatic afferent nerve although the exact underlying mechanism remains obscure. Further exploration of these concepts is warranted.

There are some limitations to this study. AgRP works as an endogenous antagonist on the melanocortin receptor. However, we could not observe the target brain site of AgRP in Evening feeding-induced insulin resistance. In addition, it was unclear how Evening feeding-induced AgRP increased TG content in skeletal muscle. In addition, it has been reported that not only TG but other lipids like diacylglycerol (DAG) and ceramide also interfere with glucose metabolism [45]. Moreover, previous studies suggest that ceramides, particularly, play a greater role in blocking insulin signals via JNK pathway than DAGs [46]. Therefore, we speculate that ceramide is one of the candidates responsible for the development of insulin resistance in skeletal muscle. However, in this study we could not separately measure these lipids. Furthermore, we did not elucidate why glucocorticoids are involved in AgRP expression. In this study, AgRP mRNA expression in the Evening group was increased at ZT8, when the mice are thought to be satiated. This suggests that the observed glucocorticoid-induced AgRP expression is not meant to stimulate feeding behavior. Since there are a few studies regarding the involvement of AgRP in emotional behaviors [47,48,49,50], we speculate that evening feeding-induced AgRP may regulate emotional behavior. Further studies are needed to verify these speculations.

## 4. Materials and Methods

### 4.1. Animals

We used 8- to 10-week-old C57BL/6 male mice (Japan SLC; Shizuoka, Japan). All experiments were approved by the Tokushima University Animal Study Committee and conducted following the Guidelines for the Care and Use of Animals approved by the Council of the Physiological Society of Japan. The animals were individually housed and maintained at 25 °C in a breeding facility on a 12 h light/dark cycle set for light on at 19:00 and off at 7:00. Mice were acclimated to this light-dark cycle for 2 weeks before prior to the experiment. Experimental animals of similar body weight were randomly assigned to one of the three groups. As shown in Figure 1A, the animals had ad libitum access to food during either the entire active period (Zeitgeber Time [ZT]12–24, Ad-lib group), the first 4 h of the active period (ZT12–16, Morning group), or the last 4 h of the active period (ZT20–24, Evening group). These feeding conditions were maintained for 2–8 weeks.

### 4.2. Cre-LoxP System

Floxed GR mice (B6.Cg-Nr3c1tm1.1Jda/J) and AgRP neuron-specific Cre-recombinase mice (Agrptm1[cre]Lowl/J) were purchased from The Jackson Laboratory (Bar Harbor, ME, USA). The floxed GR mice have loxP sites flanking exon 3 and are indistinguishable from WT littermates. Breeding these mice with AgRP-specific Cre-recombinase mice generates offspring in which GR is selectively deleted in AgRP-producing neurons.

### 4.3. Western Blotting

Mice received i.p. injections of insulin (1 U/kg) or 0.9% saline and were euthanized 20 min later by cervical dislocation. Tissues were extracted and immediately snap-frozen in liquid nitrogen. Homogenized tissue samples were subjected to sodium dodecyl sulfate-polyacrylamide gel electrophoresis (SDS-PAGE) and transferred to polyvinylidene fluoride membranes. After blocking with 5% milk dissolved in Tris-buffered saline containing 0.05% Triton X-100 (TBS-T), the membranes were incubated with anti-phospho-Ser473 Akt, anti-Akt, anti-phospho-Thr183/Tyr185 JNK/SAPK, anti-JNK/SAPK, anti-StAR antibody, anti-Cyp11A1, anti-FAS, anti-α-tubulin, or anti-β-actin antibodies (Cell Signaling Technology; Beverly, MA, USA) (1:1000) overnight at 4 °C. The next day, the membranes were washed with TBS-T and incubated with appropriate horseradish peroxidase (HRP)-conjugated secondary antibody (1:2000). After washing with TBS-T, the blots were developed using an Immobilon chemiluminescent HRP substrate (Merck Millipore; Darmstadt, Germany). The blot images were scanned and analyzed using Image J software (National Institutes of Health; Bethesda, MD, USA).

### 4.4. Immunohistochemistry

The mice were deeply anesthetized using ketamine (100 mg/kg) and xylazine (25 mg/kg). They were then transcardially perfused with 0.9% saline followed by 4% paraformaldehyde in phosphate-buffered saline (PBS, pH 7.4). The brains were removed and subsequently divided into three blocks. Brain blocks, including the hypothalamic area, were post-fixed for 1 day at 4 °C using the same fixative solution and then immersed in 20% sucrose in PBS (pH 7.4) for 3 days at 4 °C for cryoprotection. The brain blocks were sectioned coronally at 30 μm thickness using a cryostat (Leica CM1850, Leica Microsystems, Tokyo, Japan). The sections were rinsed thrice for 10 min in PBS and then incubated in 3% donkey serum containing 0.3% Triton X-100 in PBS (PBST) for 2 h at room temperature. The sections were then sequentially incubated with a primary antibody mixture containing rabbit anti-GR (1:1000, Cell Signaling Technology, #12041) and rat anti-AgRP (1:200, MO15105 Neuromics, Edina, MN) for 3 days at 4 °C. The sections were washed thrice for 15 min in PBS and then incubated with a mixture of donkey anti-rabbit immunoglobulin G (IgG) Alexa488 (1:500, Life Technologies) and donkey anti-rat IgG Alexa594 secondary antibodies (1:500, Life Technologies) in PBST for 2 h at room temperature. After washing with PBS, the sections were mounted on glass slides and coverslipped using Fluor Save Reagent (Calbiochem, Merck Millipore, Darmstadt, Germany). Brain sections were observed using a fluorescent microscope (Leica DM4000B, Leica Microsystems).

### 4.5. Quantitative Real-Time PCR

RNA was prepared from the mouse hypothalamus using an RNAiso Plus isolation kit (Takara Bio; Shiga, Japan). cDNA was generated using a Gene Amp RNA PCR kit (Applied Biosystems; Foster City, CA). We used pre-designed, gene-specific SYBR Green probes and primer sets to assess the expression levels of the following genes: *Npy* (for NPY) (forward: 5′-CACCAGACAGAGATATGGCAAG-3′; reverse: 5′-GGAAAAGTCGGGAGAACAAG-3′), *Pomc* (for POMC) (forward: 5′-AGTGCCAGGACCTCACCA-3′; reverse: 5′-CAGCGAGAGGTCGAGTTT G-3′), *Agrp* (for AgRP) (forward: 5′-GCGGAGGTGCTAGATCCACA-3′; reverse: 5′ –AGGACTCGTGCAGCCTTACAC-3′), *Hcrt* (for orexin) (forward: 5′- GGCACCATGAACTTTCCTTC-3′; reverse: 5′-GACAGCAGTCGGGCAGAG- 3′), *clock* (for CLOCK) (forward: 5-’ GAGAGCGCGAAGGAAATCT-3′; reverse: 5′-CCAATCAACAACGCC TGTAA-3′), *Arntl* (for Bmal1) (forward: 5-’GCCCCACCGACCTACTCT-3′; reverse: 5′-TGTCTGTGTCCATACTTTCTTGG-3′), *per2* (for Per2) (forward: 5-’ GTTCCAGGCTGTGGATGAA-3′; reverse: 5′-GGCGTCTCGATCAGATCCT-3′), *cry2* (for Cry2) (forward: 5-’ GCAGAGCCTGGTTCAAGC-3′; reverse: 5′-GCCACTGGATAGTGCTCTGG-3′), and *Actb* (for β-actin) (forward: 5′-CTAAGGCCAACCGTG AAAAG-3′; reverse: 5′-ACCAGAGGCATACAGGGACA-3′). The real-time (RT)-PCR reaction was carried out using an Applied Biosystems 7900HT RT-PCR thermocycler system and SYBR Green PCR Master Mix (Roche Diagnostics; Indianapolis, USA) following the manufacturer’s instructions. Gene expression levels were normalized to those of β-actin, which was used as an internal control.

### 4.6. ITTs

ITTs (1 unit/kg; Sigma Chemical Co., St. Louis, MO, USA) were performed on mice after a 4 h fast. Blood samples were drawn from the tail vein 0, 20, 40, 60, and 90 min after insulin injection. We then measured blood glucose concentrations (One Touch Ultra, Lifescan, Johnson & Johnson, New Brunswick, NJ, USA).

### 4.7. GTT

GTT (1.5 g/kg) were performed on mice after 20-h fast. Blood samples were drawn from the tail vein 0, 15, 30, 60, and 120 min after intraperitoneal injection of glucose. We then measured blood glucose concentrations (One Touch Ultra, Lifescan, Johnson & Johnson, New Brunswick, NJ).

### 4.8. Brain Cannula Implantation

Mice were anesthetized using an i.p. injection of ketamine (100 mg/kg) and xylazine (10 mg/kg). A double-walled stainless steel cannula (Unique Medical; Osaka, Japan) was then implanted stereotaxically into the right lateral ventricle according to a mouse brain atlas [51]. The stereotaxic coordinates for the lateral ventricle were 0.3 mm anterior, 1.0 mm lateral, 2.5 mm ventral to the bregma. The cannula was firmly anchored to the skull using acrylic dental cement. After the surgery, the mice were allowed 1 week of recovery to ensure body weight, and food intake returned to baseline before the experiments.

### 4.9. Antisense Oligonucleotides

All antisense oligonucleotides used were vivo-morpholinos (Gene Tools, LLC., Philomath, OR, USA). The antisense sequence used for *Agrp* was 5′-TGCAGCAGAACTTCTTCTGCTCGGTCTGCAGTTGTCTTCTTGAGG-3′ (27). The 5-missense oligo was used as the control oligo. Vivo-morpholinos were injected into the bilateral arcuate hypothalamus twice weekly, as previously reported [52].

### 4.10. Adrenalectomy

Bilateral adrenalectomy was performed via a dorsal approach under ketamine/xylazine anesthesia, as previously reported [53]. The dorsal skin was shaved and disinfected, and an incision of approximately 1 cm was made above and parallel to the spinal cord. The adrenal glands were removed from the surrounding fat tissue through small openings in the muscle layer to the left and right of the spinal cord. After the adrenalectomy, the mice were provided with corticosterone (Sigma-Aldrich, St. Louis, MO, USA) dissolved in polyethylene glycol 400 containing 5% ethanol using an osmotic mini-pump (1002, ALZET, Cupertino, CA, USA) to maintain appropriate corticosterone levels [54].

### 4.11. Microarray

DNA microarrays were used as previously described [55]. Briefly, 2 weeks into the feeding paradigms, the mice in the Ad-lib, Morning, and Evening groups were sacrificed for DNA microarray analysis at ZT8. The hypothalamus was dissected from the brain immediately after decapitation. Hypothalamic tissues were stored in RNAlater stabilization reagent (Qiagen; Hilden, Germany) for 2 h and then processed for molecular analyses (microarray and RT-PCR). Total RNA was isolated following Qiagen’s RNA isolation protocol (Rneasy Mini kit, Qiagen, Hilden, Germany). Contaminating DNA was removed using Rnase-free Dnase set (Qiagen) during the RNA purification process. The quality of the purified RNA used for the microarray analysis was assessed using an Agilent 2100 Bioanalyzer and an RNA 6000 Nano Labchip kit (Agilent Technologies, Inc.; Palo Alto, CA, USA). Equivalent amounts of total RNA from the hypothalamic of all mice in a group were mixed uniformly and then subjected to microarray analysis. Data processing was performed using Gene Spring 7.3 software (Agilent Technologies, Santa Clara, CA, USA). 

### 4.12. TG Content

TGs were extracted from skeletal muscle and livers using chloroform/methanol precipitation. Triglyceride content in the extracted lipids was measured using a commercial kit (TG E-test, Wako, Japan). 

### 4.13. NE Turnover

NE turnover was measured as the decline in tissue NE content after inhibiting catecholamine biosynthesis using α-methyl-p-tyrosine (α-MT), as described previously [12,56]. AgRP was injected into the lateral ventricle. This was followed by an injection of α-MT (200 mg/kg, i.p.) (Sigma). At 0 or 4 h after the α-MT injection, the animals were decapitated and tissues were rapidly removed and weighed. Tissue samples were homogenized in 0.2 M perchloric acid 0.1 mM ethylenediaminetetraacetic acid, the homogenates were centrifuged at 4 °C, and the NE content of the resulting supernatants was assayed using high-performance liquid chromatography (EP-300 system; Eicom; Kyoto, Japan) with a reversed-phase column (CA-5ODS, Eicom; Kyoto, Japan) and an electrochemical detector (ECD-300, Eicom). 

### 4.14. VO_2_ and RQ

VO_2_ and RQ were measured using a respiratory gas analyzer (ARCO-2000 mass spectrometer; ARCO system; Chiba, Japan). The mice were allowed to acclimatize in individual metabolic cages for 48 h before any measurements were taken.

### 4.15. Plasma Concentrations

We used commercial kits to measure plasma concentrations of glucose (Glucose CII-test, Wako, Osaka, Japan), TGs (TG E-test, Wako), non-esterified free fatty acids (NEFA C-test, Wako, Osaka, Japan), total bile acids (TBA-test, Wako, Osaka, Japan), total cholesterol (cholesterol E-test, Wako, Osaka, Japan), insulin (Insulin ELISA; Shibayagi; Gunma, Japan), and corticosterone (YK240; Yanaihara Institute, Inc.; Shizuoka, Japan).

### 4.16. Statistical Analysis

Values are expressed as the mean ± standard error in the text and figures. Data were analyzed using one-way analysis of variance. If a statistically significant effect was identified, post hoc analyses (Bonferroni or Dunn) were conducted to correct multiple comparisons. Data from two groups were analyzed using the Student’s *t*-test. *p* Values under 0.05 were considered statistically significant.

## 5. Conclusions

In conclusion, the present study demonstrated that feeding in the late active period induces whole-body insulin resistance through muscle TG accumulation and reduced insulin sensitivity via the glucocorticoids-hypothalamic AgRP pathway.

## Data Availability

The data presented in this study are available on request from the corresponding author.

## Supplementary Materials

The following are available online at https://www.mdpi.com/article/10.3390/ijms221910831/s1.
