# Supplementary material for: Feeding Rhythm-Induced Hypothalamic Agouti-Related Protein Elevation via Glucocorticoids Leads to Insulin Resistance in Skeletal Muscle
Source: Int J Mol Sci. 2021 Oct 7;22(19):10831. doi: 10.3390/ijms221910831 (PMC8509554; doi:10.3390/ijms221910831)
Supplement: Supplementary file 1 [file ijms-22-10831-s001.zip › ijms-1380478-supplementary.pdf]

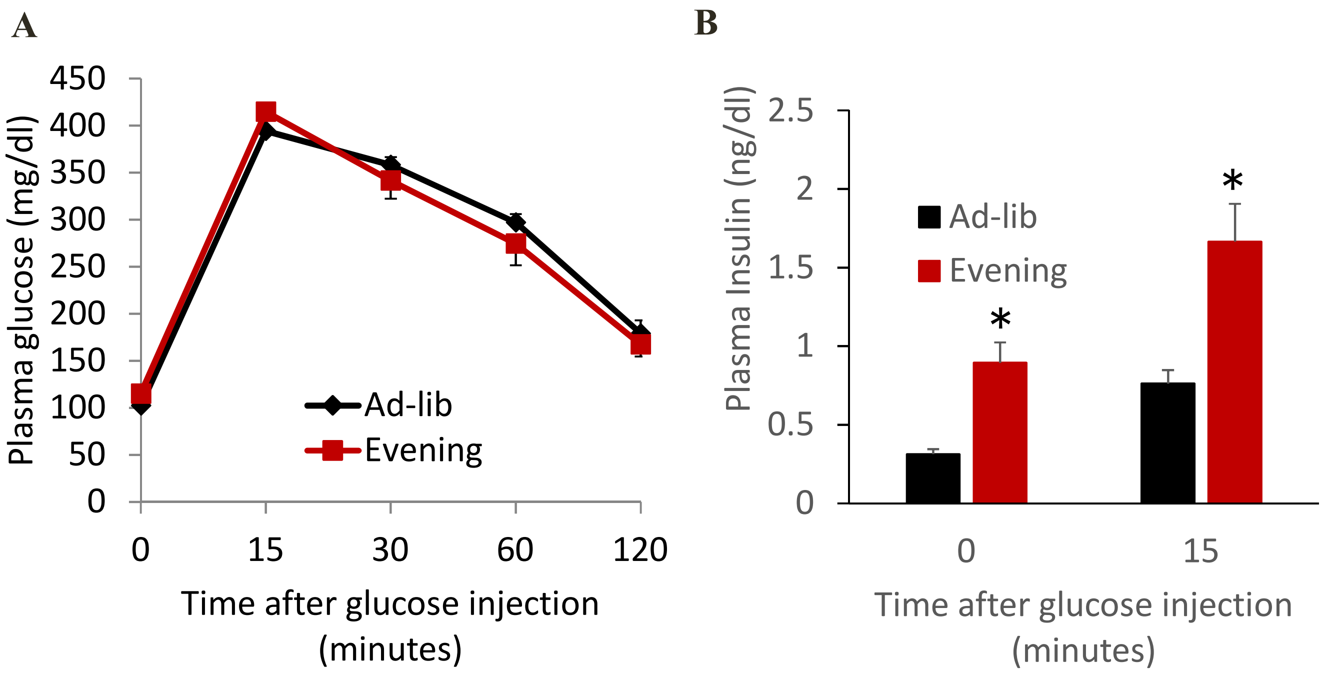

Figure S1. Glucose tolerance test. (A) plasma glucose and (B) plasma insulin concentration after intraperitoneal glucose injection. Data are presented as mean ± SE, n = 6. * $p < 0.05$ vs. Ad-lib.

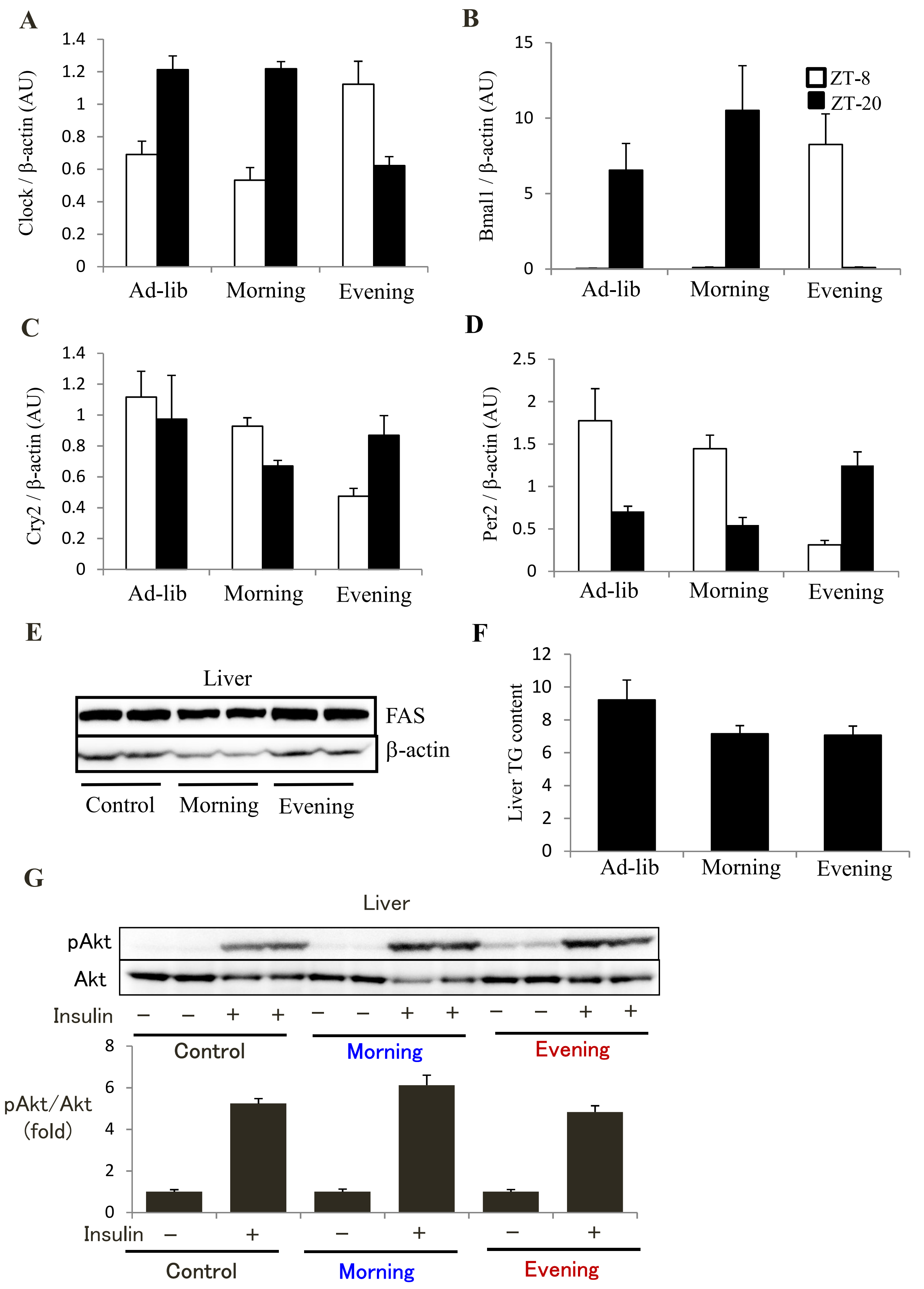

Figure S2. Effect of feeding rhythm on gene expression and metabolism in the liver. Hepatic levels of (A) clock, (B) bmal1, (C) cry2 and (D) per2 gene mRNAs, (E) FAS protein, and (F) triglyceride (TG). Data are presented as mean ± SE, n = 3-4.

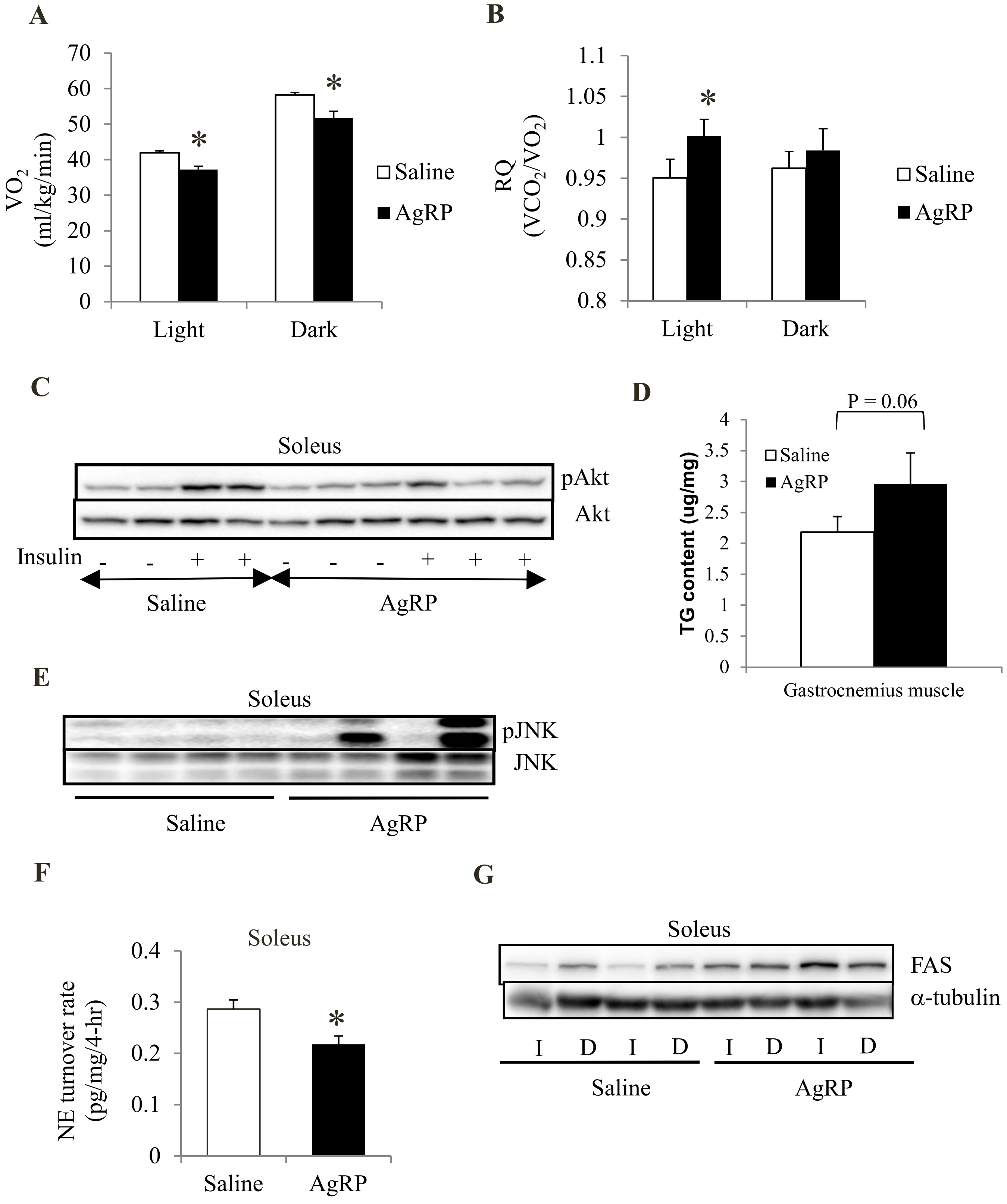

Figure S3. Effect of intracerebroventricular AgRP injection on energy metabolism. (A-E) Effect of consecutive injections of AgRP for 7 days into the lateral ventricle on muscle (A) VO2, (B) RQ, (C) insulin-induced Akt phosphorylation in muscle, (D) TG content, (E) and JNK phosphorylation. (F) Norepinephrine turnover in the soleus was decreased by a single injection. (G) AgRP injections for 1 week enhanced FAS expression in the intact soleus but not the denervated soleus, which already had enhanced FAS expression. I: intact, D: denervated.
Data are presented as mean ± SE, n = 3-6. * $p < 0.05$ vs. saline.

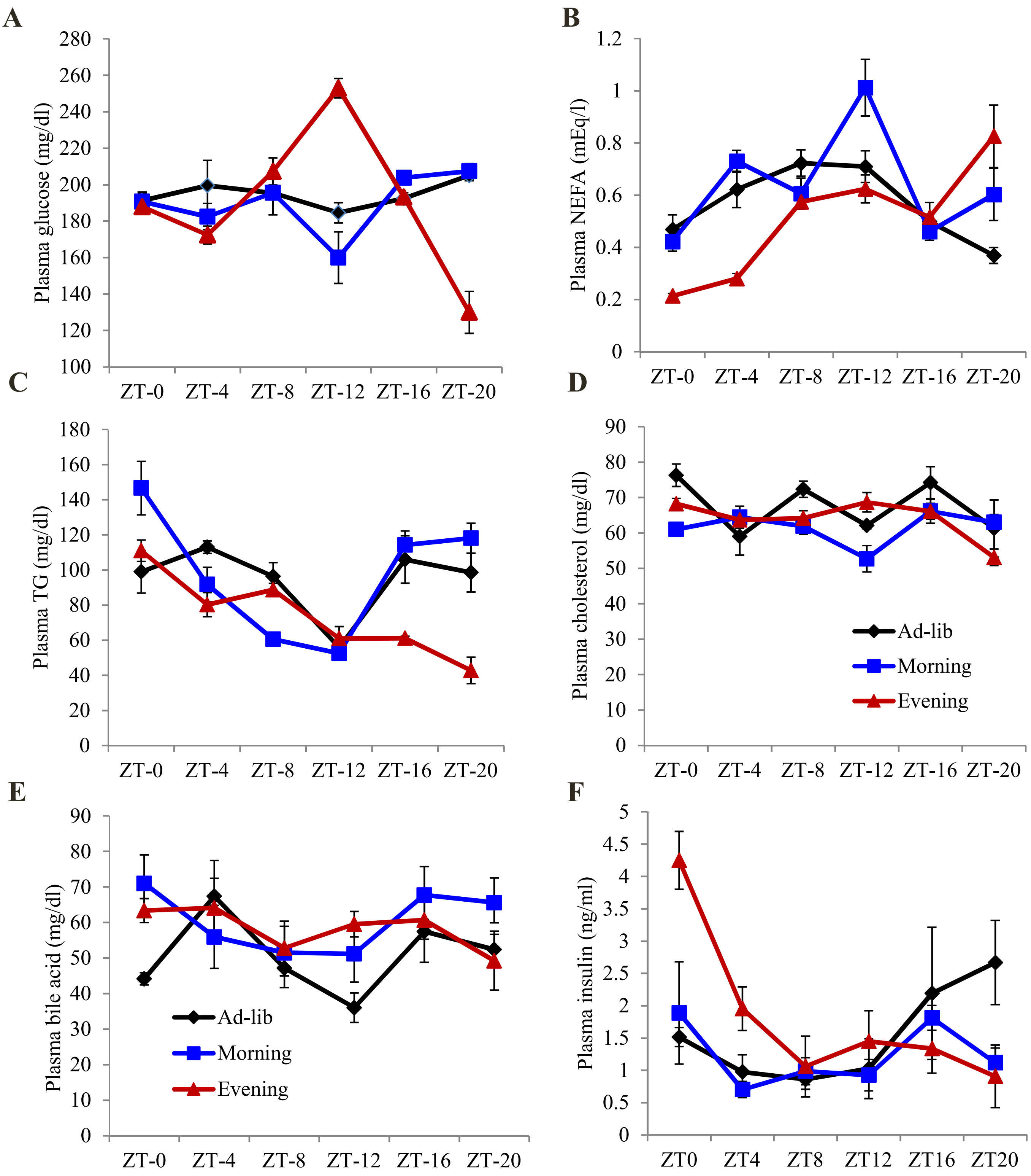

Figure S4. Circadian alterations in plasma levels of (A) glucose, (B) non-esterified free fatty acid (NEFA), (C) triglycerides (TGs), (D) cholesterol, (E) bile acid, and (F) insulin. Data are presented as mean ± SE, n = 3-4.

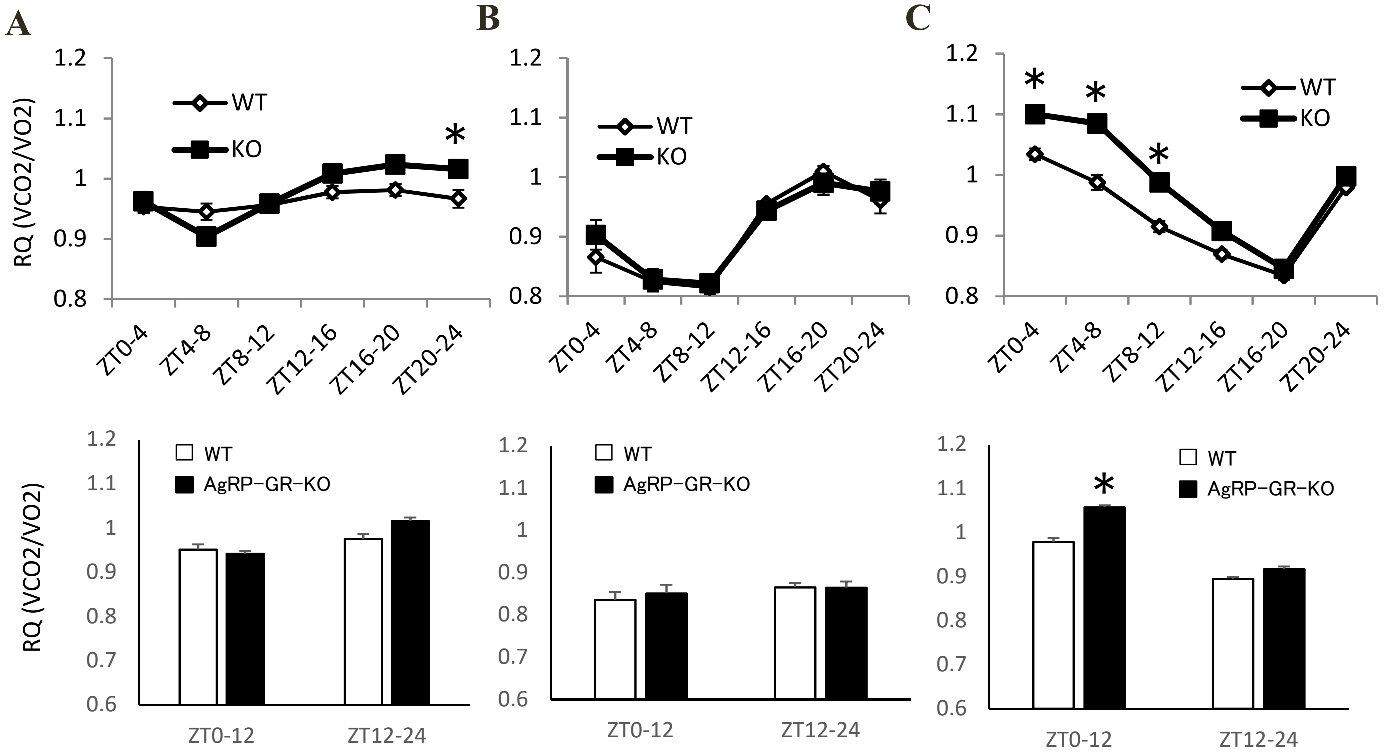

Figure S5. RQ in (A) Ad-lib, (B) Morning, and (C) Evening group of each genetic mice. Data are presented as mean ± SE, n = 3-4. * $p < 0.05$ vs. WT.

Table S1. DNA microarray performed on the hypothalamus at ZT8 in the three groups.
“GeneSymbol” indicates genes with expression levels more than 2-fold higher in the Evening group than both in the Morning and Ad-lib groups.

ProbeName	FC (abs) ([EVE] vs [Cont])	Regulation ([EVE] vs [AL])	GeneSymbol	Description
A_55_P2049717	3.3991318	up	Agrp	Mus musculus agouti related protein (Agrp), mRNA [NM_007427]
A_55_P2419514	3.2506838	up	Ccny	Mus musculus cyclin Y (Ccny), mRNA [NM_026484]
A_55_P1960896	2.7681437	up	Fndc3c1	Mus musculus fibronectin type III domain containing 3C1 (Fndc3c1), mRNA [NM_001007580]
A_55_P1965101	2.7377224	up	Xlr3b	Mus musculus X-linked lymphocyte-regulated 3B (Xlr3b), mRNA [NM_001081643]
A_51_P256827	2.6146643	up	S100a8	Mus musculus S100 calcium binding protein A8 (calgranulin A) (S100a8), mRNA [NM_013650]
A_55_P1959683	2.569272	up	Xlr3b	Mus musculus X-linked lymphocyte-regulated 3B (Xlr3b), mRNA [NM_001081643]
A_55_P1998471	2.5382109	up	S100a9	Mus musculus S100 calcium binding protein A9 (calgranulin B) (S100a9), mRNA [NM_009114]
A_55_P1985001	2.5039809	up	Cirbp	cold inducible RNA binding protein [Source:MGI Symbol;Acc:MGI:893588] [ENSMUST00000054666]
A_51_P495780	2.4072618	up	Plin4	Mus musculus perilipin 4 (Plin4), mRNA [NM_020568]
A_66_P111049	2.3629282	up	Prlh	Mus musculus prolactin releasing hormone (Prlh), mRNA [NM_001101647]
A_55_P2107239	2.2761972	up	Ggn	Mus musculus gametogenetin (Ggn), transcript variant 3, mRNA [NM_182696]
A_55_P2039416	2.2469215	up	Sgk1	Mus musculus serum/glucocorticoid regulated kinase 1 (Sgk1), transcript variant 1, mRNA [NM_001161845]
A_55_P2005475	2.1452727	up	Sult1a1	Mus musculus sulfotransferase family 1A, phenol-preferring, member 1 (Sult1a1), mRNA [NM_133670]
A_55_P2015541	2.1046102	up	Hif3a	Mus musculus hypoxia inducible factor 3, alpha subunit (Hif3a), transcript variant 2, mRNA [NM_016868]
A_51_P308347	2.0785656	up	Dact2	Mus musculus dapper homolog 2, antagonist of beta-catenin (xenopus) (Dact2), mRNA [NM_172826]
